# Expression of Prostacyclin-Synthase in Human Breast Cancer: Negative Prognostic Factor and Protection against Cell Death *In Vitro*

**DOI:** 10.1155/2015/864136

**Published:** 2015-07-27

**Authors:** Thomas Klein, Jens Benders, Friederike Roth, Monika Baudler, Isabel Siegle, Martin Kömhoff

**Affiliations:** ^1^Department of Pediatrics, Philipps University, 35033 Marburg, Germany; ^2^F. Hoffmann-La Roche, 4070 Basel, Switzerland; ^3^Dr. Margarete Fischer Bosch Institute for Clinical Pharmacology, 70376 Stuttgart, Germany; ^4^University Medical Center Groningen, 9700 RB Groningen, Netherlands

## Abstract

Endogenously formed prostacyclin (PGI_2_) and synthetic PGI_2_ analogues have recently been shown to regulate cell survival in various cell lines. To elucidate the significance of PGI_2_ in human breast cancer, we performed immunohistochemistry to analyze expression of prostacyclin-synthase (PGIS) in 248 human breast cancer specimens obtained from surgical pathology files. We examined patients' 10-year survival retrospectively by sending a questionnaire to their general practitioners and performed univariate analysis to determine whether PGIS expression correlated with patient survival. Lastly, the effects of PGI_2_ and its analogues on cell death were examined in a human breast cancer cell line (MCF-7) and a human T-cell leukemia cell line (CCRF-CEM). PGIS expression was observed in tumor cells in 48.7% of samples and was associated with a statistically significant reduction in 10-year survival (*P* = 0.038; *n* = 193). Transient transfection of PGIS into MCF-7 cells exposed to sulindac increased cell viability by 50% and exposure to carbaprostacyclin protected against sulindac sulfone induced apoptosis in CCRF-CEM cells. Expression of PGIS is correlated with a reduced patient survival and protects against cell death *in vitro*, suggesting that PGIS is a potential therapeutic target in breast cancer.

## 1. Introduction

Epidemiological studies have shown that regular intake of nonsteroidal anti-inflammatory drugs (NSAIDs) is associated with a reduced incidence of a range of epithelium-derived malignancies [1]. NSAIDs inhibit the enzymatic activity of cyclooxygenase (COX), the enzyme that provides prostaglandin H_2_ (precursor to prostacyclin [PGI_2_]) and is therefore considered to provide the rate-limiting step during prostanoid synthesis [2]. Two isoforms exist: the constitutive COX-1 and the inducible COX-2. Specific inhibitors of the latter (also called coxibs or COX-2 selective NSAIDs) have been developed because gastrointestinal side-effects of NSAIDs are thought to result from COX-1 inhibition. As COX-2 is expressed in the majority of human cancers, including breast cancer [3, 4], COX-2 selective inhibitors (coxibs) next to COX-2 unselective ones (conventional NSAID) are tested for their antitumor activity.

The most significant effects of NSAIDs have been observed in cancers of the digestive tract, including the colon [5]. The potential effect of NSAIDs in the chemoprevention of breast cancer is being investigated; however, current understanding is less clear than in colon cancer. A study in rats found a reduced relative risk of breast cancer associated with the use of coxib celecoxib [6]. Results in women are conflicting: a cohort study in women, which analyzed the incidence of breast cancer, did not find a protective effect linked to the intake of aspirin [7]. A beneficial effect of NSAIDs on the incidence of breast cancer has however been demonstrated in recent meta-analyses [8–10].* In vitro*, incubation of NSAIDs with human breast cancer cell lines has been shown to induce apoptosis [11].

In contrast to the extensively studied function of COX-2 in tumor formation, little information exists on the role of prostanoid forming enzymes and receptors acting downstream of COX. Prostacyclin-synthase (PGIS) has recently been implicated in the regulation of cell survival and induction of NSAID-mediated cell death in HT29 colon cancer cells can be abrogated by the addition of carbaprostacyclin, a synthetic analogue of PGI_2_ [12]. When exposed to hypertonic stress, cell death in rabbit renal cells was significantly reduced by addition of carbaprostacyclin but not by other prostanoids [13]. In contrast, overexpression of PGIS, as well as exogenously added carbaprostacyclin, induces apoptosis in human embryonic kidney cells (HEK293) [14]. Prostacyclin activates the adenylate cyclase coupling prostacyclin-receptor (IP), which mediates the anti-aggrgatory effect of PGI_2_ on platelets as well as its vasodilatory action on smooth muscle cells [15]. However, the effects of PGI_2_ on cell survival are independent of cyclic adenosine monophosphate (cAMP) generation and thus activation of IP. Mounting evidence suggests that PGI_2_ can activate the nuclear transcription factor peroxisome proliferator activated receptor *β*/*δ* (PPAR *β*/*δ*). Various fatty acids including carbaprostacyclin have been suggested to activate PPAR *β*/*δ* [16]. Activation of PPAR *β*/*δ*-reporter plasmids by cotransfection of PGIS indicate that, in addition to carbaprostacyclin, endogenously formed PGI_2_ may also be a ligand for PPAR *β*/*δ* [14, 17]. However, in a more recent study, endogenous PGI_2_ was not confirmed as a PPAR *β*/*δ* agonist [18]. Taken together, previous studies suggest that PGI_2_ can regulate cell survival possibly by activation of PPAR *β*/*δ*. Therefore, that PGIS could be a target in tumor biology.

In order to elucidate a potential role of PGIS in breast cancer, we analyzed the expression of this enzyme in human breast cancer and retrospectively examined its effect on patient survival. Furthermore, the effect of overexpressing PGIS on NSAID-induced cell death was studied in a breast cancer cell line (MCF-7); and the effect of the synthetic analogue carbaprostacyclin was tested similarly in a human T-cell leukemia cell line (CCRF-CEM).

## 2. Materials and Methods

### 2.1. Case Selection and Histopathology

Cases of patients with breast cancer (*n* = 248, surgery performed 1986–1990) were retrieved from the surgical pathology files of the Robert Bosch Krankenhaus (Stuttgart, Germany) and followed up with a questionnaire sent to their general practitioner. The drop-out rate was 55 patients (22.1%) without further selection or bias in the remaining 193 cases. The mean follow-up time was 67.4 months (median: 55 months [range: 1–119]). Tumor staging was performed according to World Health Organization guidelines [19]. All patients had initially undergone either mastectomy surgery or a breast-conserving resection of their primary carcinomas. We discriminated ductal invasive carcinoma (78.2%), lobular invasive carcinoma (8.8%), and invasive carcinoma specified otherwise (13.0%). Estrogen and progesterone receptor expression was analyzed biochemically with charcoal and dextran using 20 fmol/mg protein as cutoff point [20]. In addition to reviewing pathology reports, slides of all cases were reexamined for uniform assignment of grade and stage and other histopathologic features. Only the invasive tumor component was considered for evaluation.

### 2.2. Immunohistochemistry

Sections were cut (3 *μ*m thickness), deparaffinized in xylene, and incubated for 30 minutes in methanol containing 0.3% H_2_O_2_ to block endogenous peroxidase activity. Sections were then incubated with rabbit anti-PGIS polyclonal antibodies, as described previously [21]. Briefly, sections were microwaved in phosphate buffered saline (PBS) containing 0.1 M sodium citrate and primary antibodies were incubated overnight at room temperature. Immunolabeling was detected using a biotinylated rabbit anti-goat antibody followed by visualization with an avidin-biotin horseradish peroxidase labeling kit (Vectastain ABC kit) and diaminobenzidine staining. The specificity of the polyclonal antibodies for PGIS used in this study has been extensively characterized in our previous study analyzing routinely formalin fixed human tissue cut in serial sections where identical staining patterns could be demonstrated for PGIS immunoreactive protein and mRNA using immunohistochemistry and radioactive* in situ* hybridization (ISH), respectively [21]. Expression of PGIS immunoreactive protein in human breast cancer samples was analyzed independently by two investigators who were blinded to patient data. In tumor tissues, staining intensity was scored visually as absent (0), weak (1), moderate (2), or strong (3). The percentage of PGIS-positive tumor cells was graded as absent (0), 1% to 10 (1), 11% to 50% (2), 51% to 80% (3), and 81% or more (4). The immunoreactive score (IRS) index was calculated as the product of the two values [22]. Photomicrographs were viewed with a Leitz RMB microscope and pictures were captured with a digital camera (Spot-Cam, Diagnostic Instruments, Sterling Heights, MI). Color composites were generated by using Adobe Photoshop v5.0 on a Power Macintosh.

### 2.3. Cell Culture

MCF-7 human breast carcinoma cells and CCRF-cells were obtained from DMSZ (Hannover, Germany). MCF-7 cells were cultured in Dulbecco's modified Eagle's medium (DMEM) tissue culture medium supplemented with 10% (v/v) fetal bovine serum (FBS) and streptomycin and penicillin. CCRF-cells were grown in Roswell Park Memorial Institute (RPMI) medium 10% FBS supplemented with gentamycin. Cultures were incubated at 37°C in 95% O_2_ and 5% CO_2_. Tissue culture medium was changed every 48–72 hours.

### 2.4. Generation and Functional Characterization of a Prostacyclin-Synthase Expression Vector

A murine full length PGIS cDNA was amplified from total neonatal kidney cDNA using Advantage two-step polymerase chain reaction (Clontech, CA). The PGIS upstream primer was 5′CTTGTTGCCACCCTGCAGCC 3′, and the downstream primer was 5′CAGGAAGTCAGAAGGCCCCA 3′. DNA-fragments were cloned into pCDNA 3.1 expression vector (Invitrogen, Nl) to yield pCDNA3.1mPGIS. An enzymatically inactive mutant of mPGIS (PGIS C441A) was prepared by site directed mutagenesis (QuikChange, Stratagene, CA) according to Hatae and coworkers [23]. Oligonucleotide primers used to prepare the mutants were 5′-AGG GCA CAA CCA GAG CCT GGG GAA GAG TTA TGC C-3′ and 5′- GGC ATA ACT CTT CCC CAG GCA CTG GTT GTG CCC T-3′. Expression of wild-type and mutant PGIS was analyzed by Western blot analysis using the same rabbit polyclonal antibodies as for the immunohistochemical analysis as described previously [24]. Briefly, 20 *μ*g of total cell lysates was harvested 48 hours after transfection, separated on 10% SDS-PAGE, blotted onto nitrocellulose, and probed with the rabbit polyclonal anti-PGIS antibodies (diluted 1 : 500). Enzymatic activity of wild-type PGIS protein was shown by detection of 6-keto-prostaglandin F1*α* (6-keto-PGF_1*α*_), the stable metabolite of PGI_2_, by gas-chromatography/mass spectrometry (GC/MS) in supernatants from transfected cells, as described previously [25]. Cotransfection for 48 hours of a COX-2 expression vector (pCDNA3.1COX-2, kindly provided by Dr. Guan et al. [26], with pCDNA3.1mPGIS (wild-type PGIS)) into MCF-7 cells resulted in abundant generation of 6-keto-PGF_1*α*_ (1.48 ± 0.286 ng/mL of supernatant). No significant generation of 6-keto-PGF_1*α*_ was observed in MCF-7 cells coexpressing pCDNA3.1COX-2 with either pCDNA3.1 or pCDNA3.1mPGISC441A (0.03 ± 0.009 and 0.0023 ± 0.01 6-keto-PGF_1*α*_ ng/mL, resp.).

### 2.5. Transfection of cDNA and Experimental Design

All plasmid-mediated transfections were performed on 40–60% confluent cells using Polyfect (Qiagen, Germany). pCDNA3.1mPGIS and pCDNA3.1COX-2 (2 *μ*g each) were transfected into 6-well dishes. Cells were then transferred to DMEM medium supplemented with 0.5% (v/v) FBS for 24 hours and exposed to 150 *μ*M sulindac and sulindac sulfone for additional 24 hours. Cell viability was assessed using the 3-(4,5-dimethylthiazol-2-yl)-2,5-diphenyltetrazolium bromide (MTT) assay.

The effect of the stable PGI_2_ analogue carbaprostacyclin on cell viability was analyzed in CCRF-CEM cells exposed to sulindac sulfone. Cells were grown in RPMI supplemented with 10% (v/v) FBS and gentamycin. CCRF-CEM cells were coincubated for 48 hours with sulindac sulfone (100 and 300 *μ*M) and either vehicle, increasing concentrations of carbaprostacyclin (0.01–1 *μ*M), or the membrane permeable cAMP analogue dibutyryl-cAMP (dbcAMP, 0.001–10 mM). Apoptotic cell death was analyzed by measuring caspase-3 activity as assessed by cleavage of Ac-DEVD-AMC fluorogenic substrate (Pharmingen, CA).

### 2.6. Statistical Methods

Patient data assessments were conducted using SPSS (SPSS Software GmbH, Munich, Germany). Survival curves were established according to the Kaplan-Meier method, and comparisons between survival curves were performed with the log-rank test. Overall survival was calculated from the date of surgery to death or to the date of the last patient contact. Disease-free survival was measured from the date of surgery until the time of relapse, cancer-related death, or last contact. Patients who died from unrelated causes were considered censored by the time of their death. To define a cutoff point for PGIS expression, the minimal *P* value approach was applied. The IRS ≥3 was used for all further analyses. Multivariate analyses were performed using Cox regression analysis in a model with T, N, M, G, and ER and PR status. Association between PGIS expression and other parameters such as age, tumor size, nodal status, and hormonal status was assessed by the test.

Cell culture experiments were analyzed with Student's *t*-test.

## 3. Results

### 3.1. Expression of Prostacyclin-Synthase in MCF-7 Cells

**Figure 1:**
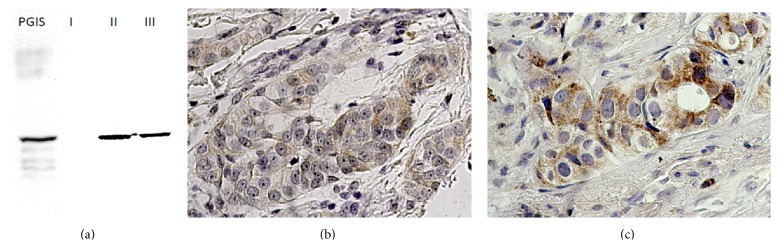
Immunological detection of prostacyclin-synthase. (a) Western blot analysis of MCF-7 cells transfected with control vector (lane I: pCDNA 3.1), wild-type (lane II: pCDNA3.1mPGIS), and mutant prostacyclin-synthase (lane III; pCDNA3.1 mPGISC441A). As positive control PGIS from bovine aorta is shown on the left. ((b), (c)) Expression of immunoreactive PGIS in tumor cells of a ductal carcinoma showing moderate (b) or intense labeling (c). Slides were photographed at 63x magnification.

To confirm expression of wild-type and mutant PGIS protein, Western blot analysis was performed using MCF-7 cell lysates (20 *μ*g per lane). In Figure 1(a), a band could not be detected in cells transfected with the control vector pCDNA 3.1 (lane I). Bands of approximately 52 kD corresponding to the molecular weight of PGIS could be detected in cells transfected with both the PGIS wild-type vector pCDNA3.1mPGIS (lane II) and the mutant PGIS vector pCDNA3.1 mPGISC441A (lane III). Purified PGIS from bovine aorta endothelial cell (left lane) served as positive control.

### 3.2. Expression of Prostacyclin-Synthase in Breast Cancer Tissue

Expression of PGIS immunoreactive (ir) protein was examined in tumor samples from 248 patients with breast cancer obtained at diagnosis of primary breast cancer disease. Patient age was 26–86 years; median (±SD) age was 56.49 ± 12.11 years. PGIS-immunoreactivity in tumor cells was observed in 48.7% of samples and was generally weak in tumor cells. In PGIS-positive tumor cells, cytoplasm and perinuclear staining was observed consistent with the expression of PGIS in the endoplasmic reticulum and the perinuclear envelope [27]. Expression of PGIS ir-protein in tumor cells differed in both staining intensity and percentage of positive tumor cells (Figures 1(b) and 1(c)). PGIS-immunoreactivity was also observed in various cell samples known to express this enzyme (fibroblasts: 68%; inflammatory cells: 62.2%; and vessels 61.7%; data not shown).

### 3.3. Univariate Analysis

**Figure 2:**
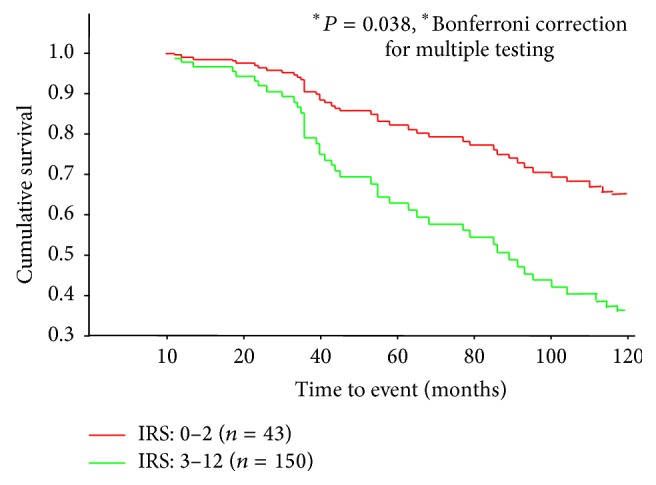
Relationship between PGIS expression and overall survival of 193 patients. The Kaplan-Meier survival curves shown are for subgroups with either low (IRS2) or high (IRS ≥ 3) PGIS expression among all patients under study.

Kaplan-Meier survival curves were created from the data of 193 patients with complete information (see Table 1) to evaluate the prognostic value of established parameters for overall survival. As expected, the classical prognostic factors, that is, histology grade, tumor size, and nodal status, were all significantly associated with overall survival, whereas age, steroid receptor status, and menopausal age were not (Table 1). To evaluate a possible relationship between PGIS expression and disease outcome, different IRS subgroups were initially defined and Kaplan-Meier analysis was performed for overall survival. In these analyses, it became apparent that subgroups with higher PGIS expression levels had shorter mean survival times than subgroups with lower expression (data not shown). This suggested the use of a single cutoff value to simplify further analyses. To select a value, the minimal *P* value approach was used, and cutoffs from IRS 1 to 5 were compared by Kaplan-Meier analysis. The statistical results, with and without application of the Bonferroni correction for multiple statistical testing, are listed in Table 2. The most discriminative value (IRS ≥ 3) was used for further subgroup analyses and multivariate Cox regression analysis. At 10 years following their diagnosis, 64.6% of patients with low PGIS expression (IRS < 3) were still alive compared with 36.4% in the group with high PGIS expression (IRS ≥ 3, Figure 2).

### 3.4. Multivariate Analysis

On multivariate Cox analysis, the following factors were tested: tumor size, nodal status, tumor grading, and PGIS expression (cutoff IRS ≥ 3). In this model, PGIS expression did not prove to be an independent prognostic factor.

### 3.5. Overexpression of PGIS in MCF-7 Cells

The role of PGIS in the protection against NSAID-induced cell death was examined in human MCF-7 breast cancer cells. In vehicle-treated (0.1% DMSO) cells viability was not affected by transfection with wild-type or mutant PGIS vector (compared to control vector, data not shown). Upon exposure to sulindac (150 *μ*M) for 24 hours, the viability of cells transfected with control vector and cells transfected with mutant PGIS was significantly reduced compared with MCF-7 cells transfected with wild-type PGIS as assessed by the MTT assay (mock: 0.153 ± 0.19 and PGISC441A: 0.2065 ± 0.038 compared with wild-type PGIS: 0.312 ± 0.048 optical density 540 nm/690 nm). In contrast to mock- and pCDNA3.1PGISC441A-transfected cells, overexpression of wild-type PGIS also increased cell viability in MCF-7 cells exposed to 150 *μ*M sulindac sulfone compared with, albeit to a lesser extent than with, sulindac (data not shown).

### 3.6. Effect of Carbaprostacyclin on Cell Viability in CCRF-Cells

**Figure 3:**
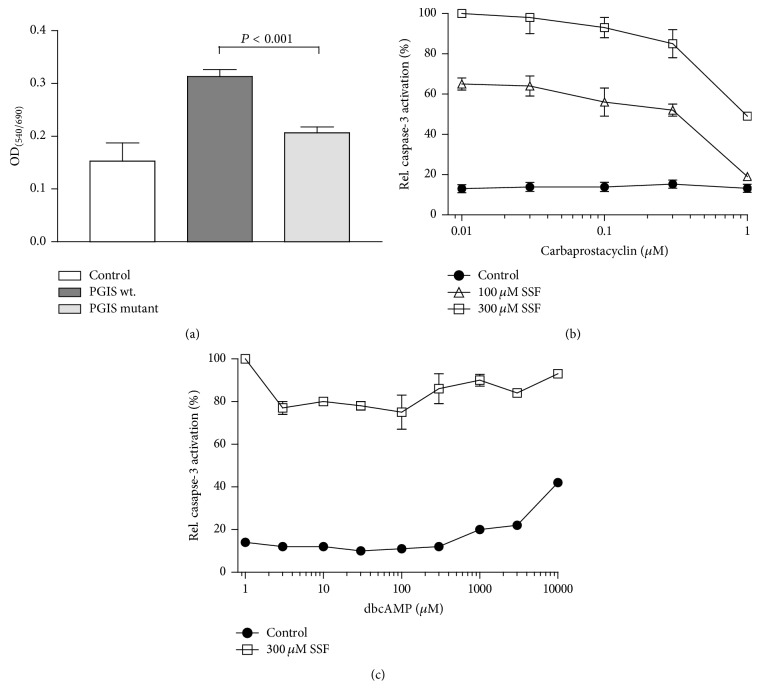
Effects of overexpression of PGIS and carbaprostacyclin on cell survival. (a) MCF-7 cells were transiently cotransfected with pCDNA3.1COX-2, together with control vector pCDNA3.1, pCDNA3.1mPGIS, and pCDNA3.1mPGISC441A. Cell viability upon exposure to 150 *μ*M sulindac for 24 hours was examined by the MTT assay as compared to vehicle-treated controls (0.1% DMSO). ((b), (c)) Carbaprostacyclin-mediated protection from sulindac sulfone-induced apoptosis. CCRF-CEM cells were treated with 0, 100, and 300 *μ*M sulindac sulfone in the presence of (b) 0.01–1 *μ*M carbaprostacyclin or (c) 0.001–10 mM dbcAMP. 24 hours posttreatment caspase-3 activity was measured by cleavage of fluorogenic substrate Ac-DEVD-AMC.

Exposure of CCRF-CEM cells to 100 and 300 *μ*M sulindac sulfone induced apoptotic cell death in a dose-dependent manner as analyzed by measurement of caspase-3 activity (Figure 3(b)). Coincubation of CCRF-CEM cells with sulindac sulfone and increasing concentrations of carbaprostacyclin (0.01–1 *μ*M) resulted in a decrease of apoptotic cells by about 50% at either sulindac sulfone concentration and by carbaprostacyclin 1 *μ*M. Thus, treatment with the synthetic PGI_2_ analogue carbaprostacyclin protected against NSAID-induced apoptosis. Cells were coincubated with sulindac sulfone and the membrane permeable cAMP analogue dibutyryl-cAMP (dbcAMP) to rule out involvement of the classical prostacyclin-receptor IP in protection against apoptotic cell death via elevation of intracellular cAMP. Cell viability was not affected upon treatment with dbcAMP suggesting a mode of action independent of IP modulation (Figure 3(c)).

## 4. Discussion

To address directly the potential role of PGIS in cell survival, we analyzed the effects of PGIS-overexpression in a human breast cancer cell line MCF-7 and of carbaprostacyclin treatment in a human immature T-cell line CCRF-CEM cells. MCF-7 cells overexpressing PGIS showed a significant increase in cell viability when challenged with sulindac and sulindac sulfone. Given that sulindac inhibits prostaglandin-production, and thus PGI_2_ formation, we assume that PGI_2_ generated during the first 24 hours after transfection and prior to the addition of sulindac rendered the cells resistant to the adverse effects of sulindac (sulfone).

Apoptotic cell death of sulindac sulfone treated CCRF-CEM cells exposed to increasing concentrations of carbaprostacyclin was also significantly reduced. To exclude an activation of the classical IP pathway, CCRF-CEM cells were treated with a stable cell permeable cAMP analogue (dbcAMP). The lack of effect of this agent argues against a role of the IP-receptor in the protection against apoptosis.

To the best of our knowledge this is the first study to investigate the expression of PGIS in primary human breast cancer. Data currently available are conflicting. One investigation found no PGIS expression in human lung carcinoma [28], whereas another described a significant reduction of PGIS protein expression in non-small cell lung cancers [29]. However, no correlation with overall patient survival was observed. A more recent study used the same antibody (anti-PGIS) to investigate expression in head and neck squamous cell carcinoma [30]. Lower PGIS levels were observed in tumor samples than in nontumoral mucosa. Patients who expressed high levels of PGIS in head and neck squamous cell carcinoma had a higher 5-year survival rate than the low PGIS expressing group.

Our present study using an ISH-validated polyclonal antibody [21] on mamma CA also showed weak immunoreactivity in tumors; however, our striking results from retrospective analysis revealed that expression of PGIS is associated with a reduction of patient survival. Although statistical significance was not achieved, the data indicated that patients with a high IRS had a worse prognosis than patients with a low IRS. Given the paucity of data addressing the expression and roles of PGIS and its putative receptor PPAR *β*/*δ* in human breast cancer a variety of mechanisms can be postulated that link PGIS expression with a reduction in patient survival. Expression of PGIS in human breast cancer cells might increase their viability* in vivo* resulting in a less favorable prognosis than for patients who lack PGIS expression in their cancerous cells. This is supported by data that showed that the PPAR *β*/*δ* ligand, cPGI, protects HT29 colon cancer cells against cell death* in vitro *[12]. Likewise, cPGI has been shown to rescue renal medullary interstitial cells from cell death [13]. Finally, activation of PPAR *β*/*δ* protected cultured murine keratinocytes against cell death [31]. Importantly, the contention that PGI_2_ promotes survival of breast cancer cells is compatible with the data from several meta-analyses that demonstrated the chemopreventive effect of NSAIDs on the formation of breast cancer in women [8–10]. Inhibition of PGI_2_ formation by NSAIDs would neutralize the stimulatory effect of PGI_2_ in malignant cells, leading to a reduced incidence of breast cancer.

Alternatively, PGI_2_ might actually reduce cell viability as shown by a study in which endogenously expressed PGI_2_ promoted apoptosis in human embryonic kidney cells and Caco-2 colon cancer cells [14]. This might indicate that administration of NSAIDs to patients expressing PGIS might actually be disadvantageous. This notion is not supported by epidemiological data, although patient survival has not been stratified in these patients in terms of PGIS expression. These apparently contradictory actions of PGI_2_ on cell survival may indicate that its effects are highly dependent on the specific cellular environment.

Despite the fact that the effects of cPGI and PGIS on cell survival are clearly divergent, there is agreement that these effects are not dependent on cAMP generation (and thus activation of IP) but are possibly mediated by PPAR *β*/*δ* [14, 17, 31–33]. In contrast to PPAR *γ* the potential roles of PPAR *β*/*δ* have yet to be studied in mammary cancer cells. Two studies addressed the role of PPAR *β*/*δ* in colon cancer, one demonstrated that xenografts of null cells (PPAR *β*/*δ* ^−/−^ cells) derived from the human colon cancer cell line HCT 117 exhibited a significant reduction in tumor formation compared with wild-type HCT 117 cells (PPAR *β*/*δ* ^+/+^ cells) [34]. However, the essential role of PPAR *β*/*δ* for intestinal tumor formation could not be confirmed in another study using PPAR *β*/*δ* knockout mice; polyp size, but not polyp number, was reduced in PPAR *β*/*δ*-null mice compared with wild-type mice [35]. More recently, we published data showing impaired tumor-angiogenesis in PPAR *β*/*δ*-null mice [36].

Little information exists on the specific role of PGI_2_ in human breast cancer. One study showed that elevated levels of the PGI_2_ metabolite, 6-keto-PGF_1*α*_, in breast cancer tissue are associated with a more aggressive phenotype [37]. In rats, inhibition of thromboxane-synthase by imidazole led to enhanced cancer multiplicity in an N-methyl-N-nitrosourea induced breast cancer model. In contrast, administration of tranylcypromine which inhibits PGIS has been shown to reduce cancer multiplicity, indicating that inhibition of this enzyme, but not thromboxane-synthase, might be useful in the chemoprevention of breast cancer [38].

## 5. Conclusions

We have shown that expression of PGIS in human breast cancer is a negative prognostic factor. Overexpression of PGIS increases cell viability in MCF-7 cells exposed to sulindac and sulindac sulfone and carbaprostacyclin protects against sulindac induced apoptosis in CCRF-CEM cells. The apparent discrepancy to the inverse correlation of PGIS expression and survival in other carcinomas (e.g., head and neck tumors) could not only be explained by hormonal biases. More and larger epidemiological studies in different tumors are needed to analyze the importance of PGIS expression as an independent prognostic factor. The products and molecular targets of PGIS and their implication in mammary tumor formation need to be further elucidated to investigate whether certain subgroups of breast cancer patients show different survival rates in relation to PGIS expression.

## Figures and Tables

**Table 1:** Statistics on patients' clinical data, classical prognostic factors, and PGIS expression (*n* = 193).

Parameter	*N* (193)	PGIS expression				*P*
		IRS: 0*–*2	%	IRS: 3*–*12	%	
		Patients (*N*)		Patients (*N*)		
Median age, years		54				
Age *≷* median		83/110				0.127^*∗*^
<56 years	83	66	79.5	17	20.5%	>0.05^*∗∗*^
>56 years	110	84	76.4	54	23.6%	
Menopausal status						
Pre/post/?	56	37/100/56				0.715^*∗*^
Premenopausal	37	30	81.1	7	18.9%	>0.05^*∗∗*^
Postmenopausal	100	81	81.0	19	19.0%	
Tumor size						
T1/T2/T3/T4/?	4	29/102/45/8/9				0.0068^*∗*^
<2 cm	45	39	86.7	6	13.3%	>0.05^*∗∗*^
>2 cm	144	109	75.7	35	24.3%	
Nodal status						
N0/N1/N2/N3/?	3	80/92/13/5/3				<0.0001^*∗*^
N0 node negative	80	63	78.8	17	21.3%	>0.05^*∗∗*^
N1–N3 node positive	110	85	77.3	25	22.7%	
Grading						
G1/G2/G3/?	4	8/122/59/4				0.0402^*∗*^
G1 & G2	130	108	83.1	22	16.9%	>0.05^*∗∗*^
G3	59	39	66.1	20	33.9%	
ER/PR						
++//+−&−+//−−//?		96/30/57/10				0.230^*∗*^
Pos/pos	96	78	81.2	18	18.8%	>0.05^*∗∗*^
Pos/neg or neg/pos	30	20	66.6	10	33.3%	
Neg/neg	57	43	75.4	14	24.6%	

PGIS = prostacyclinsynthase; IRS = immunoreactive score; IRS 0–2 = low PGIS expression; IRS 3–12 = high PGIS expression; *N* = number; cm = centimeter; ER = estrogen receptor; PR = progesterone receptor; pos = positive; neg = negative.

^*∗*^ *P* value for overall survival (log-rank test).

^*∗∗*^ *P* value for expression of PGIS (*χ* ^2^ test).

**Table 2:** Kaplan-Meier overall survival analysis for different immunoreactive scores.

Cutoff (IRS)	Number	Overall survival prognosis	
		Log-rank *P*	Corrected *P* ^*∗*^
≥1	99/94	5.56	0.0184
≥2	129/64	4.37	0.0377
≥3	150/43	8.37	0.0038
≥4	161/32	6.35	0.0117
≥5	177/16	4.50	0.0338

IRS = immunoreactive score, ^*∗*^Bonferroni correction for multiple testing.

## Abbreviations

NSAIDs: Nonsteroidal anti-inflammatory drugs
COX: Cyclooxygenase
PGI_2_: Prostacyclin
PGIS: Prostacyclin-synthase
HEK293: Human embryonic kidney cells
IP: Adenylate cyclase coupling prostacyclin-receptor
cAMP: Cyclic adenosine monophosphate
PPAR *β*/*δ*: Peroxisome proliferator activated receptor *β*/*δ*
MCF-7: Human breast cancer cell line
CCRF-CEM: Human T-cell leukemia cell line
PBS: Phosphate buffered saline
ISH: * In situ* hybridization
IRS: Immunoreactive score
DMEM: Dulbecco's modified Eagle's medium
FBS: Fetal bovine serum
RPMI: Roswell Park Memorial Institute
6-Keto-PGF_1*α*_: 6-Keto prostaglandin F1*α*
cDNA: Complementary deoxyribonucleic acid
GC/MS: Gas-chromatography/mass spectrometry
MTT: 3-(4,5-Dimethylthiazol-2-yl)-2,5-diphenyltetrazolium bromide assay
dbcAMP: Dibutyryl-cAMP
ir: Immunoreactive.
